# Modelling the effect of gravity on inert‐gas washout outputs

**DOI:** 10.14814/phy2.13709

**Published:** 2018-05-20

**Authors:** Brody H. Foy, Sherif Gonem, Chris Brightling, Salman Siddiqui, David Kay

**Affiliations:** ^1^ Department of Computer Science University of Oxford Oxford Oxfordshire United Kingdom; ^2^ Respiratory Biomedical Research Centre University of Leicester/National Institute of Health Research Leicester Leicestershire United Kingdom

**Keywords:** Body position, computational modelling, gravity, Inert‐gas washout, pulmonary function testing

## Abstract

Multiple‐breath washout (MBW) is a pulmonary function test (PFT) that is used to infer lung function through measurement of ventilation heterogeneity (VH). However, the body position that a test is taken in may also influence VH, due to the “Slinky” effect of gravity on the lungs. In healthy subjects this has minimal effect, but in unhealthy groups, PFT outputs have been seen to change drastically with body position. In this study, we used a combined computational and clinical approach to better understand the response of outputs from the MBW to body position. A patient‐specific model of the MBW was developed, then validated against clinically measured washout data, as well as broader results in the literature. This model was then used to compare changes in MBW outputs with respect to body position, showing that output changes sensitively predict regional airway size differences between lobes. We then highlight cases in which body position effects may bias MBW outputs, leading to elevated or masked responses to bronchoconstriction. We close by placing this result in context with broader clinical practice, and showing how it can help improve interpretation of test outputs.

## Introduction

The conducting zone of the human lungs typically consists of 16‐17 generations of bifurcating airways, subtending into approximately 130 000 terminal bronchioles. Distally, the left and right main bronchus bifurcate, and feed into 5 distinct lobes of uneven size. In diseases such as asthma, inflammation and mucus secretion cause the conducting airways to narrow, leading to strongly heterogeneous flow. Clinically, this flow heterogeneity is measured through at‐the‐mouth pulmonary function tests (PFTs) such as spirometry, oscillometry and inert‐gas washouts (Pellegrino et al. 2005; Marchal and Hall 2010; Robinson et al. 2013). It is well known in the literature that flow heterogeneity is affected by body position, being attributed to the “Slinky effect” where a lung deforms unevenly under gravitational strain (Hopkins et al. 2007). However, if flow heterogeneity is primarily driven by airway morphology (Horsfield and Cumming 1968), it may be assumed that the body position in which a test is taken under would have a relatively small effect on the test outputs.

In studies of PFT outputs under various body positions, and in micro‐gravity, relatively small changes are seen in the PFT outputs of healthy subjects (Behrakis et al. 1983; Prisk et al. 1995; Grönkvist et al. 2002; Rodríguez‐Nieto et al. 2002; Peces‐Barba et al. 2004). However, in studies of elderly or unhealthy patient groups the results are much more inconsistent, noisy, and often contradictory. This has been illustrated in clinical research studies (Attinger et al. 1956; Manning et al. 1999; Badr et al. 2002; Smith et al. 2017), as well as imaging studies (Petersson et al. 2007). Moreover, understanding of the precise way in which gravity influences lung function in unhealthy patients is still limited. Clinically, some PFT outputs have been shown to be highly sensitive to position, particularly in patients with unilateral disease (Zack et al. 1974; Prokocimer et al. 1983; Gillespie and Rehder 1987). Equally, for some clinical practices, such as mechanical ventilation, there is disagreement over which position should be used (Thomas and Paratz 2007; Fessler and Talmor 2010), partially stemming from a lack of precision in prior studies of how gravity interacts with morphology in the context of disease.

We hypothesize that significant differences in test outputs taken from different positions predominantly occur in patients with strongly regionalized lung disease. To test this hypothesis a combination of computational modeling and clinical data is used. First, a model of inert‐gas washout is designed, and run on patient‐specific virtual lung structures. The model is benchmarked against patient‐specific clinical measurements, and broader results from the literature, showing high accuracy at recreating patient test values, as well as recreating broader phenomena. Once benchmarked, the model is used to analyze test output changes under varying body positions, illustrating how different positions result in significantly different output values from the same patient, only if significant regionalization of bronchoconstriction is present. We close by more carefully examining results in sitting and supine positions, and discuss the potential interpretation of these results in a clinical setting.

## Methods

### Clinical data collection

Details of the collection of clinical inert‐gas washout data are outlined in a previous study by Gonem et al. (2014). The data from the subjects with asthma was derived from the baseline data of a placebo‐controlled trial of Fevipiprant (Gonem et al. 2016). In short, washouts were performed by 31 subjects (seven healthy, 24 asthmatic), in the sitting position, with SF_6_ as the tracer gas. Each washout was performed in triplicate, using current guidelines (Robinson et al. 2013) and the method described by Horsley et al. (2008). For each run, *s*_cond_ (Verbanck et al. 1997) and the Lung Clearance Index (LCI) (Horsley et al. 2008) were calculated (see Appendix 1 for definitions), with the pooled averages being presented in this study. Each subject's function residual capacity was also calculated by dilution from the washout. All washout concentrations were monitored by an Innocor photoacoustic gas analyser (Innovision A/S, Odense, Denmark).

Basic statistics of the subject group are given in Appendix 2.

### Lung structure creation

Each patient also underwent inspiratory‐expiratory CT imaging in the supine position. In a previous study, Bordas et al. (2015) segmented the CT images, to virtually extract the central airways (up to generation 6–10), then used an energy minimization algorithm (Fetita et al. 2004) to generate the remainder of the conducting zone branches. All of the algorithmically generated airways were grown within the lobar boundaries, as identified from the CT images. The resulting virtual airway structures contained between 30,000 and 120,000 branches each.

Each virtual structure also had its degree of disease regionalization classified, by comparing mean small airway sizes at various locations. If the mean small airway size in the left lung was significant larger (*>*10%) than in the right lung, the patient was classified as having “Right” regionalization, and vice versa as “Left”. Similarly, if the mean small airway size in the lower lobes was significantly larger than in the upper lobes, the patient was classified as “Upper”, and vice versa as “Lower”. If no significant difference in airway size was seen, the structure was classified as having “Neither” regionalization.

### Inert‐gas washout model

Ventilation distributions in the lung structures were simulated using a model previously outlined by Foy and Kay (2017) and Foy et al. (2017), with details given in Appendix 3. In short, flow was assumed proportional to the pressure gradient over each branch, with Poiseuille flow resistance and a correction term for energy dissapation (Pedley et al. 1970). Terminal bronchioles were connected to individual acinar regions, which expanded and contracted proportional to flow rates in the bronchiole, the pressure gradient from the pleural cavity to the bronchiole, and regional tissue compliance. Total lung compliance was scaled from a standard value of 0.2L.cmH_2_O^*−*1^ (Galetke et al. 2007) per each patient's functional residual capacity (FRC) by the multiplicative factor FRC/2.5, and distributed evenly amongst the acinar regions. Pleural pressure was assumed sinusoidal in time (a simplistic approximation presented in the work of Ben‐Tal (2006)), with a breathing period of 4 sec, and tidal volume of 1L.

Gravitational effects were incorporated by enforcing a linear gradient on the pleural pressure range (see Appendix 3). Within the literature, reported ventilation and perfusion gradients typically vary in the range 1–5% cm^*−*1^ (Kaneko et al. 1966; Hopkins et al. 2007; Henderson 2013; Horn et al. 2013). For this study, we have set the gradient strength as 1.5% cm^*−*1^, so as to not over‐magnify the effect we are investigating. We note that this value was also derived from a computational study of tissue mechanics (Swan et al. 2012). Using this gravitational gradient, different body positions were simulated by modifying the direction of gravity; orthostatic (cranio‐caudal axis), supine and prone (anterior‐posterior axis) and left‐ and right‐lateral (transverse axis).

The simulated ventilation distribution (*u*) was used to drive a convection‐diffusion transport model for gas concentration (Dutrieue et al. 2000), which simulated the wash‐in and washout of *SF*_6_. This model was solved over the entire airway tree (and associated acinar regions) using a finite difference method in MATLAB. Initially the lungs were started with no gas concentration, and the model was used to simulate a wash‐in, until exhaled gas concentrations stabilized (changed by less than 0.5%) over consecutive breaths. Following this, the washout was simulated over multiple consecutive breaths, until the mean exhaled gas concentrations fell below 1/40th of the mean exhaled concentration from the first washout breath. From each washout curve, *s*_cond_ and LCI were calculated. Due to the simplicity of the respiratory zone in this model, the MBW output sacin was not simulated, as it is known to respond to diffusive effects in the respiratory zone (Robinson et al. 2013).

A brief sensitivity analysis of the model's parameters is given in Appendix 3.

### Inert‐gas washout simulations

Washouts were simulated in each of the 31 patient‐specific virtual structures, across five positions (standing, supine, prone, left‐lateral and right‐lateral), and without gravity. Each washout was performed using gas properties consistent with an *SF*_6_ washout, and a wash‐in concentration of 4%. For each simulation a ventilation heat map was also created, using the process outlined in Appendix 3.

## Results

To apply the computational model in a way that is relevant clinically, we use a multi‐step validation process. The model is first validated directly against clinical data. Following this, comparisons to broader findings in the literature are made, to validate against aspects for which there is no available structure specific clinical data. The model is then used to investigate the effects of body position on the inert‐gas washout.

### Validation against clinical data

**Figure 1:**
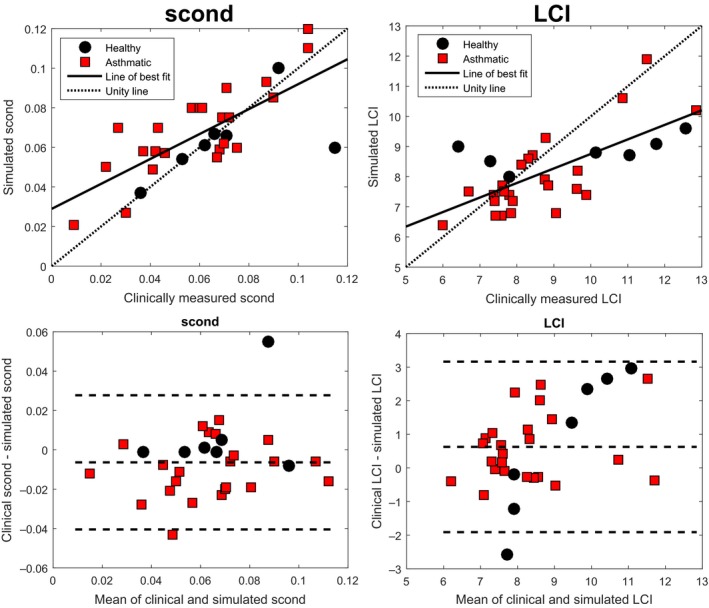
Correlation of clinical and simulated *s*_cond_ (top left) and LCI (top right) values, for healthy and asthmatic subjects. The line of best fit is overlaid to both plots, as well as the unity line. Both fits are strongly significant (*P *≪* *0.05), with *R* ^2^ values of 0.51 and 0.57, respectively. The Bland–Altman plot of each fit is also shown (bottom), with *s*_cond_ showing low bias, and constant variance (despite one significant outlier). Simulated LCI values show a small negative bias, with a potential small positive correlation.

In Figure 1 we compare simulated LCI and *s*_cond_ values (standing) against the corresponding clinical measurements. Both measures can be seen to have a significant positive correlation with clinical data, of strong statistical significance (*P *≪ 0.05), with *R* ^2^ values of 0.51 and 0.57, respectively. The Bland–Altman plots indicate that *s*_cond_ in particular is being well captured by the model, with little bias (0.005), constant variance, and the 95% confidence interval (*−*0.04*,* 0.035). LCI still maintains a good fit, but is clearly more variable than that of *s*_cond_, with larger bias (0.66), and 95% confidence interval (*−*1.95*, −*3.1). LCI potentially also has a slight positive correlation in the Bland–Altman plot.

It can be noted in Figure 1 that 4 of the healthy subjects reported quite high clinical LCI values. This was primarily due to the higher age of these subjects, as can be seen in Appendix 2.

### Validation against the broader literature

**Figure 2:**
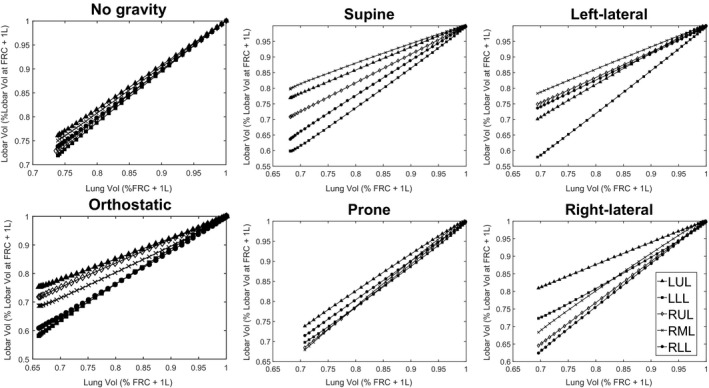
Relative ventilation of each of the five lobes during exhalation: left‐upper (LUL, triangle), left‐lower (LLL, square), right‐upper (RUL, diamond), right‐middle (RML, cross), and right‐lower lobe (RLL, circle). A flatter gradient indicates that the lobe experienced a relatively smaller change in volume over the exhalation. Data were simulated from the structure of a healthy patient.

Patient‐specific clinical data was only obtained in one body position (sitting). To validate the gravitational component of our model, we compare outputs to the broader literature. In Figure 2 we show simulated ventilation distributions for a healthy patient in the five body positions and without gravity.

The results in Figure 2 accurately reproduce the ‘Slinky’ effect, with the vertically higher lobes ventilating more heavily than the lower lobes. Panel B of the figure also creates a ventilation pattern very similar to that of Verbanck and Paiva (2016), with the right‐middle lobe changing the least during exhalation, and the lower‐lobes changing the most. We note that the scales of these exhalations are also quite similar between the two studies, with the right‐middle lobe reducing to 80% of its end‐inhalation volume by the end of exhalation in both cases. Equally, we note that the prone ventilation distribution is more homogeneous than the supine distribution, again in line with prior clinical studies (Mure et al. 2000).

**Figure 3:**
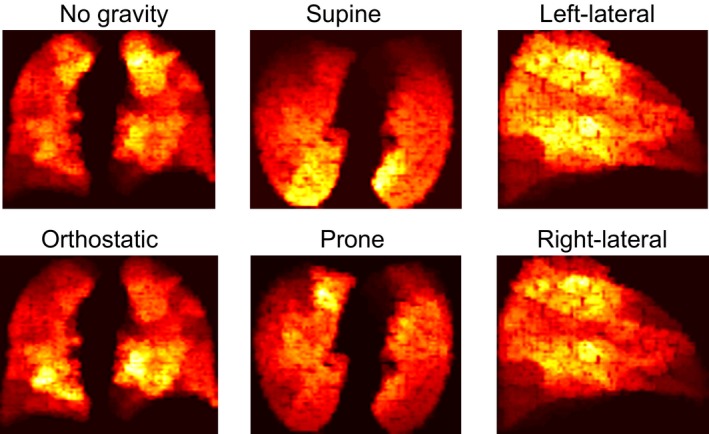
Plot of specific ventilation ratios (the ratio between volume entering a region, and end‐expiratory volume of that region) in a central 3 cm lung slice; with no gravity and orthostatic (coronal plane), supine and prone (transverse plane), and left‐lateral and right‐lateral (sagittal plane). Brighter colors indicate higher ventilation, showing the magnitude of effect that body position can have. Slices were taken directly from the centre of the lungs for the coronal and tranverse planes, and from the center of the left lung for the sagittal plane. Data were simulated from a healthy patient.

In Figure 3 we give the simulated ventilation maps corresponding to Figure 2. These results match qualitatively to results seen in MRI in the literature (Petersson et al. 2007; Hamedani et al. 2016). In particular, the supine ventilation map strikingly resembles a supine map from the work of Horn et al. (2013). The ventilation ratio gradients (relative to the mean ventilation ratio) were 4.4, 2.8, 5.7, 5.4, and 4.9% cm^*−*1^ for supine, prone, left‐lateral, right‐lateral, and orthostatic, respectively. We note that the value for supine is in line with the clinically measured value reported by Horn et al. (2013), and matches to other reported values in the literature, as does the prone gradient (Musch et al. 2002; Hamedani et al. 2016).

To interpret the results in Figures 2 and 3, care should be taken in considering the position of each lobe in different body positions. The position of the lower lobes is defined by the two oblique fissures, which are diagonal. This means that the lower lobes are lowest to the ground in both supine, and orthostatic positions. The oblique fissure is also typically more cranio‐caudal in the left lung than in the right lung. This means that in a left‐lateral position the left‐lower lobe is significantly lower than the left‐upper lobe, while in a right‐lateral position, the difference between right‐lower and right‐upper lobe is less pronounced. Finally, both lungs are larger toward the back than the sternum. This, combined with the positioning of the oblique fissures, means lobe volumes are more even toward the sternum than the back, leading to a more flat ventilation distribution in prone. We note that all of these phenomena are exhibited in Figures 2 and 3.

### Predicting lung disease regionalization through position changes

We hypothesize that MBW outputs exhibit consistent and predictable changes as body position is altered, and that these changes are driven by regionalization in the patient's underlying lung morphology. To test this hypothesis, we predict regionalization (as classified in the methods section), based on the change in test indices from opposing body positions. We expect MBW outputs to be healthiest (lowest) when taken from a body position that best ventilates the most constricted region. Considering the Slinky effect, we can expect each position to best ventilate the most gravitationally low region. Thus, if LCI (supine) is significant greater (*>*10%) than LCI (prone), we take this as predictive of “Upper” classification (as supine best ventilates the lower lobes). We make similar predictions for “Lower”, “Left”, “Right”, and “Neither”, using both LCI and *s*_cond_.

**Figure 4:**
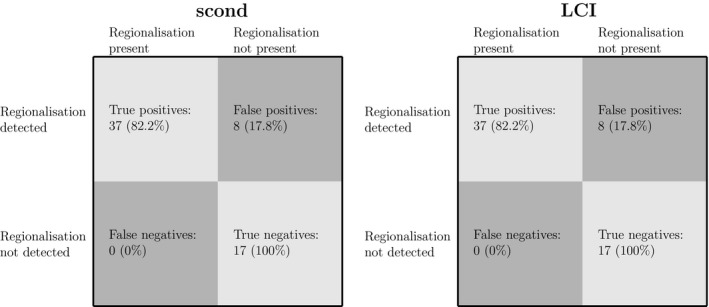
Sensitivity of *s*_cond_ and LCI changes as detectors of bronchoconstriction regionalization. Data for lateral and vertical regionalization has been combined. Note that both *s*_cond_ and LCI achieve high true positive rate, and perfect true negative rates.

In Figure 4 we give the accuracy of using MBW output changes to classifying airway regionalization. As the results show, the LCI and *s*_cond_ are highly sensitive predictors, with strong accuracy, high true positive/negative rates, and low false positive/negative rates. This suggests that the response of the MBW outputs to changes in body position are consistent, being largely driven by the underlying airway morphology.

## Discussion

In this study, we have developed a computational model of multiple‐breath washout, suitable for simulation on patient‐specific virtual lung structures. We have benchmarked this model against clinical data, showing strong patient‐specific correlations of test outputs; and illustrated its ability to recreate broader results from the literature. We have then used this model to investigate function‐form relationships between body position and test‐outputs in a unique and novel way.

### MBW dynamics

Considering Figures 1, 2, 3, we note that the correlation between the model and clinical data (both specific and broader) is quite strong, and that simulated *s*_cond_ and LCI both have quite low bias, and reasonable confidence intervals for error. The Bland–Altman plots show that *s*_cond_ in particular appears to be well captured by the model. The higher variability of response in LCI may be due to the simplifying assumptions in the model, which would lead to an underestimation of flow resistance, and subsequently a reduction in LCI in severe disease. Considering the healthy subjects, there is also a slight positive correlation. This is partially due to the influence of age, with older subjects producing higher LCI values, potentially due to changes in compliance, which the model did not account for.

However, the strength of correlation should be interpreted in reference to the fact that the virtual structures rely on algorithmic generation to create the small airways. The only patient‐specific information given to the model for each simulation is the airway structure (branch lengths, angles, radii), and the subject FRC. This highlights the mechanics that the MBW is most strongly responding to. The virtual structures are physically accurate to generations 6–10, relying on algorithmic generation and extrapolation of airway radii beyond this point. Despite the algorithmic component, the asthmatic structures were shown to have significantly higher total resistances, as well as smaller and more varied homothety ratios (in the segmented airways) (Bordas et al. 2015). Given the simple respiratory zone approximation in the model, this suggests that the response of LCI and *s*_cond_ are most dominantly driven by central and medial airway sizes, and subsequent flow rates, or that the response to small airway sizes is more statistical than specific in nature. Clearly this is a question for future research.

### Prediction of airway regionalization

The results in Figure 4 show a strong and consistent response of the MBW outputs to airway size regionalization. In particular, the results suggest that gravitationally driven changes in ventilation patterns are strong enough to influence MBW outputs, particularly in unhealthy patients, who experience high degrees of ventilation heterogeneity. Given the scale of observed ventilation changes between opposing positions (Fig. 2), this is not surprising. However, the consistency of this response is noteworthy, suggesting that the effect is strong enough to reverse‐engineer for classification. This means that the response should be understood in a clinical setting, as for a patient with strong degrees of regionalization, testing in a standard body position may either mask or exacerbate the MBW response.

The response occurred consistently for both LCI and *s*_cond_. As body position is changed to preference a more constricted region, flow rates to that region will increase. In healthy subjects, flow rates into each of the lobes are already quite high, meaning the change has a negligible effect, leading to minimal changes in LCI or *s*_cond_. However, in a patient with regionalized bronchoconstriction, flow rates will be lowest in the constricted region (a phenomena which was partially investigated in our prior work (Foy et al. 2017)). Thus, a body position which preferences flow to the constricted region will lead to overall higher flow rates, and a subsequent decrease in LCI. Equally, by improving flow to the constricted region, the overall range of time constants (Otis et al. 1956) across the lung will be reduced, leading to a decrease in *s*_cond_, and a larger change in MBW outputs between positions.

The fact that body position can affect MBW outputs is not on its own a novel contribution. As early as 1966, Bryan et al. (1966) illustrated how gravity creates a pleural pressure gradient, and how this creates differences in relative ventilation of the lobes. Many studies of healthy subjects (Behrakis et al. 1983; Prisk et al. 1995; Grönkvist et al. 2002; Rodríguez‐Nieto et al. 2002; Peces‐Barba et al. 2004) have consistently shown insignificant changes in outputs, particularly once FRC changes are accounted for.

However, studies of unhealthy patient groups have shown both drastic increases and decreases, with little explanation of why (Attinger et al. 1956; Manning et al. 1999; Badr et al. 2002). One study that did outline this response more clearly was that of Zack et al. (1974), who showed a strong correlation between classification of right‐lateral lung disease, and increases in Pa_*O*2_ between left‐lateral and right‐lateral. The results that we have presented extend upon and broader this idea, providing a more strongly quantitative interpretation of the phenomena.

### Comparison of sitting and supine outputs

In clinical settings PFTs are rarely performed in lateral or prone positions, predominantly being performed either upright or in supine. Due to the diagonal nature of the oblique fissure, these two positions produce similar relative ventilation patterns (Fig. 2). This suggests that both positions have similar biases, with higher sensitivity toward bronchoconstriction in the upper lobes than in the lower lobes. This is not an inherent flaw in the MBW, or with testing in upright/supine, but rather a phenomenon that should be understood when interpreting test outputs. Equally, these biases may also be present in imaging techniques, as shown in Figure 3, and seen in prior studies in the literature.

**Figure 5:**
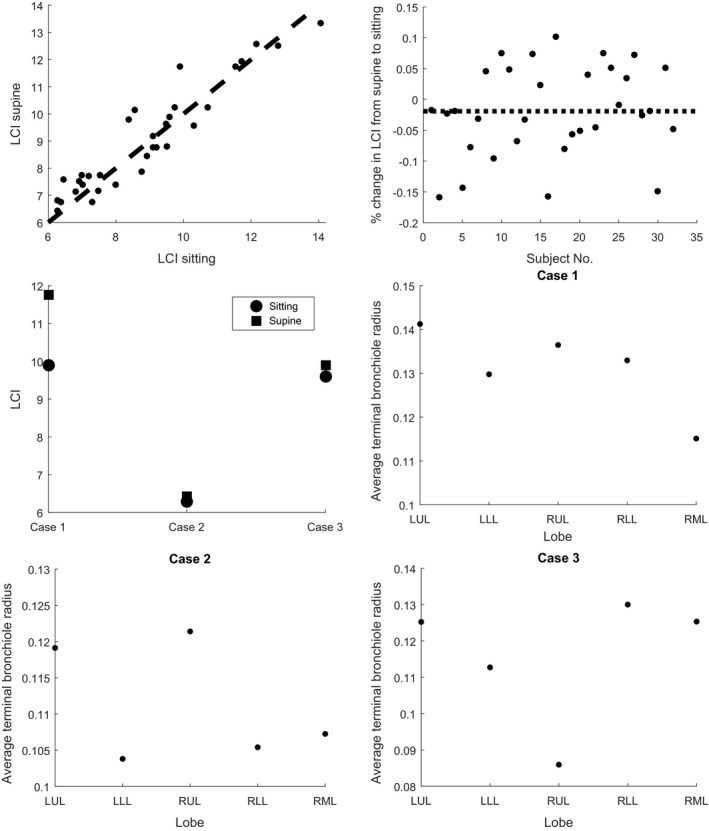
Comparison of LCI values in sitting and supine. Values are strongly, though not perfectly correlated, with an average decrease of 2% from supine to standing (top row). Specific LCI changes are highlighted in 3 cases (mid‐left), with corresponding mean airway sizes for each lobe (mid‐right, bottom row). In case 1 the right‐middle lobe is most constricted (leading to a large change in LCI), in case 2 the lower‐lobes are most constricted (leading to very little change, and a lower baseline LCI), and in case 3 the right‐upper lobe is most constricted (leading to very little change, and a higher baseline LCI). All values were simulated from patient structures within the dataset.

Furthermore, while relative ventilation is similar between supine and upright, it is not identical, with the right‐middle lobe being most significantly affected. This means that for specific disease cases, the response in MBW outputs may be quite different. We illustrate this by considering LCI changes from sitting to supine across the entire patient group, and specifically in 3 distinct cases (Fig. 5). As shown, there is a strong correlation between the two outputs, with on average a small decrease from supine to sitting, consistent with prior results in the literature (Hanson et al. 1962). However, in the case where the right‐middle lobe has the lowest small airway size, the difference in LCI from sitting to supine is quite large, as the ventilation changes most strongly affect this lobe.

### Clinical relevance

To the best of our knowledge, this is the first combined computational‐clinical study to investigate this phenomenon. Through the use of modeling, we can more precisely investigate the function‐form relationship, and give strength to the interpretation of the effect of body position. Beyond this, the results suggest the potential for clinical utility. In clinical literature there is disagreement over when different positions should be used to mechanically ventilate patients (Thomas and Paratz 2007; Fessler and Talmor 2010). The results in this study suggest that comparison of opposing position PFT outputs (or CT/MR images) could be used to classify patient groups in a clinical setting, allowing for more targeted treatment protocols. If classification was accurate enough, this could be of particular benefit in informing mechanical ventilation procedures in ARDS, or for chest clearance through physiotherapy. Clearly a targeted clinical study would be needed to investigate these hypotheses further. However, this initial study forms a strong theoretical foundation which can be built upon in future work.

### Limitations and interpretation of the study

The model outlined in this study is intended as a tool to improve understanding of the function‐form relationships involved between MBW outputs and lung morphology. While the model was strongly validated, there were a few phenomena that it did not account for, in particular these were a lack of airway dynamism throughout the breathing cycle, the assumption of static compliance, and the simplistic nature of respiratory zone structures. Each of these simplifications would lead to an underestimation of flow resistance, and in particular would have contributed to the slight negative bias of the model seen in Figure 1. Equally, the model assumed a simplistic sinusoidal pleural pressure function, which is based on prior work in the literature (Ben‐Tal 2006), but not truly representative of standard tidal breathing. The model also assumed a constant and isotropic diffusion, an assumption primarily made due to the lack of complex structural information about the respiratory zone, where gas transport is more diffusion dominated. The investigation of more complex adaptations of the model is a clear avenue for future research.

The virtual structures used in this study also rely in part on algorithmic generation of small airways. The airway sizes below generation 10 are not necessarily specifically representative of the patient, but instead an extrapolation from the airway sizes measurable from CT segmentation. This means that the models only capture airway asymmetry that originates above generation 10. Given the strong correlations of the model with clinical data, we do not believe that this represents an issue in interpretation of study results. However, the consistency in airway decrease in the lower generations may have led to a reduction in overall noise within the results, leading to stronger results than would be seen clinically.

The study was performed with a large number of asthmatics (Kaneko et al. 1966) relative to healthy subjects (Dutrieue et al. 2000), and that the age range of healthy subjects was quite large. This means that the conclusions are strongest for asthmatic subjects. However, given the wide array of literature investigating gravitational effects on ventilation in healthy subjects, we do not believe that this significantly detriments the study.

Finally, it should be noted that by its nature, the MBW does not assess regions of the lung affected by airway closure, and as such, these effects were not quantified in the current study (except briefly in the results in Figure 3).

These results are intended as a groundwork for future investigations. They suggest two major ideas. First, that MBW outputs may under or over‐predict lung disease in patients with strong disease regionalization. This means that for some patient groups, it may be more appropriate to measure lung function from non‐standard positions, to overcome this bias. Second, that information about regionalization may be gained by comparing MBW outputs from opposing positions. In future work, we aim to investigate more directly how these insights can be applied to improve clinical outcomes for patients.

### Summary

In summary, we have used both clinical and computational data to improve understanding of how MBW outputs may be affected by body position. As body position is changed the relative ventilation of each lobe is also changed, due to shifts in the gravitational ventilation gradient. This shift has minimal effect in healthy patient groups, but creates a bias that can significantly change MBW outputs in unhealthy patient groups, particularly those with disease regionalization. The effect that body position has on MBW outputs appears to be consistent and interpretable. This means that the biases of each of the positions can potentially be understood and accounted for in clinical settings.

## Conflict of Interest

CB has received research grants and consultancy from GSK AZ/MedImmune, Novartis, Chiesi, Pfizer, Vectura, Theravance, BI, Roche/Genetech.

## Figures and Tables

**Figure 6:**
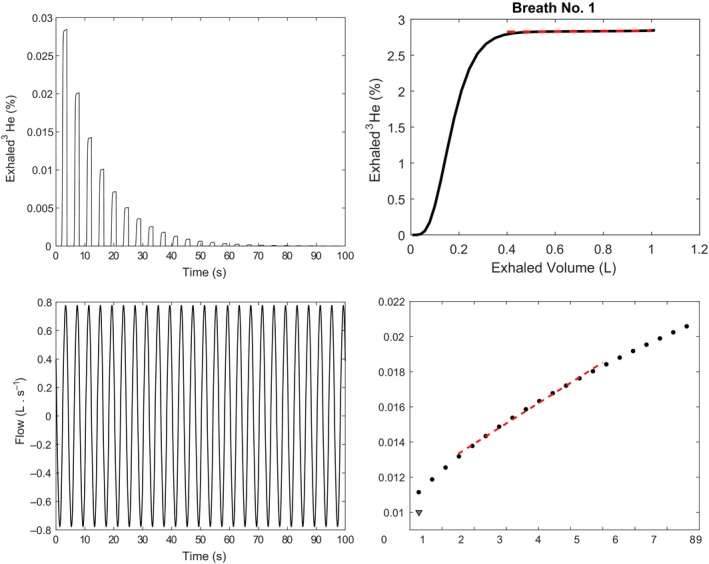
A simulated multiple breath washout curve (top left), for an artificially healthy subject. The profile for the 1st breath (right, black line) is given, with the *s*_III_ slope before normalization overlaid (gray dashed line). Inhaled volume (i.e., volume above FRC) for the test is also given (bottom right). The total set of s‐values is given (bottom left), with the slope *s*_cond_ overlaid (gray dashed line), and the index *s*_acin_ plotted (gray triangle).

## Appendix 1 Multiple breath washout indices

To calculate the major MBW indices, the expired volume, and expired gas concentration must be measured for the duration of the washout phase. The expired gas concentration is typically plotted against time, and referred to as the washout curve. An example washout curve is given in Figure 6.

The Lung Clearance Index (LCI) is defined as the time taken for the mean exhaled gas concentration over a breath to drop below 1/40th of the initial mean exhaled concentration. To normalize between subjects this time is expressed in turnovers, being defined as the cumulative expired volume divided by the individual's functional residual capacity.

To calculate *s*_cond_, we first calculate the set of *s*_III_ values between turnovers 1.5–6, which are defined as the plateau gradients of each exhalation, divided by the mean exhaled concentration over the plateau (see Fig. 6). *s*_cond_ is then defined as the mean gradient of the *s*_III_ values between turnovers 1.5–6.

## Appendix 2 Summary statistics of the subject group

Basic summary statistics of the 31 subjects in this study are given in Table A1.

**Table A1:** Clinical summary statistics of the subject group. Unless otherwise values numbers are reported as mean (std)

	Healthy (*n* = 7)	Asthmatic (*n* = 27)
Age (years)	52.6 (13.5)	50.3 (13.2)
Sex (% male)	57	64
BMI (kg m^−2^)	30 (6.32)	30.4 (5.6)
GINA (number with 0, 1–3, 4–5)	7, 0, 0	0, 5, 19
FRC (L)	2.3 (0.4)	2.5 (0.7)
LCI (Turnovers)	9.5 (2.22)	8.77 (1.74)
*s*_cond_ (L^*−*1^)	0.07 (0.02)	0.06 (0.03)

## Appendix 3 Details of the computational model

The MBW model consists of two major components; a ventilation model to calculate the flow distributions, and a gas transfer model to simulate the washout. The ventilation model simultaneously calculates flow rates (*Q*) in each branch, pressure (*P)* at each bifurcation, and the volume (*V)* of each acinar region. These variables are linked by the equation system.

ΔP=R(Q)Q,overeachbranch,

$$Q_{\mathrm{parent}}=\sum Q_{\mathrm{daughter}},\;\mathrm{over}\;\mathrm{each}\;\mathrm{bifurcation},$$

$$\frac{d}{dt}V=Q_{\mathrm{in}},\;\mathrm{at}\;\mathrm{each}\;\mathrm{terminal}\;\mathrm{branch},$$

$$P_{T}-P_{\mathrm{pl}}\left(t\right)=\frac{1}{c}V,\;\mathrm{for}\;\mathrm{each}\;\mathrm{acinus},$$

where *Q*_in_ is the flow from the terminal bronchiole into the acinus, *P*_*T*_ is the pressure at the distal end of the terminal bronchiole, *P*_pl_(*t*) is the pleural pressure outside the acinus, and C is the local compliance of the acinus. The flow resistance *R*(*Q*) is a modified Poiseuille flow term, originally presented by Pedley et al. (1970).

The temporal derivative was discretized using a Backward Euler method, and the entire system was solved in MATLAB using a vector‐Newton method, with sufficiently small time step to ensure solution accuracy.

The flow rates from the ventilation model were used to calculate velocities (*u * =  *Q/S*) in a convection‐transport model:

$$\frac{\partial C}{\partial t}=D\frac{\partial^{2}C}{\partial x^{2}}+\frac{D}{S}\frac{\partial S}{\partial x}\frac{\partial C}{\partial x}-u\frac{\partial C}{\partial x},$$

where *x* is the axial direction of flow, *t* is time, *C* is the *SF*_6_ concentration at a given location in the airway tree, *D* is the diffusion coefficient of *SF*_6_ in air (0.102 atm.cm^2^ s^*−*1^ (Marrero and Mason 1972)), and *S* is the cross‐sectional area of the branch.

The model was discretized using a Backward Euler method, and the finite difference formula outlined by Dutrieue et al. (2000). The system was solved in MATLAB using reordered LU factorization, with a sufficiently small timestep to guarantee stability. The system was started with no initial gas concentration, and an steady inflow (during inhalation) of *SF*_6_, at a concentration of 4%. During exhalation gas was removed from the trachea proportional to the tracheal flow rate. Once exhaled gas concentration stabilized over consecutive breaths the wash‐in of *SF*_6_ was halted, and the washout was simulated. The simulation was halted once peak exhaled gas concentration over a breath fell below 1/40th of the initial peak exhaled concentration, and at least six turnovers had been performed.

### Gravitational gradient

Gravitational effects were incorporated by enforcing a gradient on the pleural pressure range, such that the pleural pressure was given as:

$$P_{\mathrm{pl}}={\overset{¯}{p}}_{\mathrm{pl}}+R_{p}f\left(z\right)\sin\left(\frac{2\pi t}{T}\right).$$

where *P*_pl_ is the pleural pressure, *P*¯_pl_ and *R*_p_ are the mean pleural pressure and pleural pressure range, *T* is the breathing period, and the gravitational gradient *f*(*z*) is given as:

$$f\left(z\right)=\left(1=\frac{z-\overset{¯}{z}}{z_{\max}-z_{\min}}g_{\mathrm{strength}}\right),$$

where *z*¯*, z*_max_ and *z*_min_ are the mean, maximum and minimum heights (relative to the body position) of terminal bronchioles in the lung, and *g*_strength_ is the strength of the gradient, taken as 1.5% cm^*−*1^ (Kaneko et al. 1966).

In simulations *P*¯_pl_ and *R*_p_ were started at standard values (−10 and 10 cmH_2_O), then adjusted slightly to enforce the desired FRC and tidal volume.

Originally, more complex models based on tissue mechanics were investigated and implemented, based on the work of Swan et al. (2012). However, these were seen to introduce significant computational expense, and not significantly change the ventilation distribution.

### Ventilation maps

Following the process outlined by Foy et al. (2017), the ventilation model was used to create a series of ventilation maps, as a way to approximate MR images. In short, ventilation ratios (*r*) were calculated for each acinar region as follows:

$$r=\frac{V_{f}}{V_{f}+V_{r}},$$

where *V*_*f*_ is the fresh gas entering a region over an exhalation, and *V*_*r*_ is the residual gas in the region after exhalation. Ratios were averaged over each voxel in a 3D 80 *×* 80 *×* 80 grid, overlaid on the structure. 2D slices images were created by averaging the *r* values for each voxel in a central 3 cm slice of the lungs (in the appropriate axis). Gradients of ventilation ratios in each axis were calculated by considering the average change in *r* values over the central 3 cm slice, in the desired axis.

### Sensitivity of model parameters

Nearly all model parameters were either inferred from the patient‐specific clinical data (subject FRC, airway structure and radii), or taken from physics studies (the diffusion rate of SF_6_ in air). The four parameters which were not inferred in one of these ways were the total lung compliance, mean pleural pressure and pleural pressure range (all initially taken from mean‐population values, then slightly adjusted to enforce patient FRC and 1L tidal breathing), and the gravitational gradient strength (set as 1.5% cm^*−*1^). The sensitivity of the gravitational gradient strength (defined as the percentage change in LCI caused by an increase in gravitational strength of 1% cm^*−*1^, in a healthy patient) was calculated as approximately 4%.
